# Systemic inflammatory and oxidative stress biomarkers as predictors of pain in sequential bilateral phacoemulsification for age-related cataract

**DOI:** 10.3389/fmed.2025.1626533

**Published:** 2025-07-11

**Authors:** Peng Yin, Liyuan Wang, Yongsheng Hou, Yang Huang, Dawei Sun, Dewang Shao

**Affiliations:** ^1^China Rong Tong Medical and Healthcare Group Co. Ltd, Beijing, China; ^2^Department of Ophthalmology, Second Affiliated Hospital, Harbin Medical University, Harbin, China; ^3^Department of Ophthalmology, The First Affiliated Hospital, Harbin Medical University, Harbin, China; ^4^Department of Ophthalmology, PLA General Hospital, Beijing, China; ^5^Department of Ophthalmology, Air Force Medical Center, Beijing, China

**Keywords:** age-related cataract, phacoemulsification, pain prediction, inflammatory biomarkers, oxidative stress, patient-centered outcomes

## Abstract

**Background:**

Second-eye cataract surgery is frequently associated with enhanced pain perception compared to first-eye procedures, yet the underlying pathophysiological mechanisms remain incompletely understood. This study investigated whether systemic inflammatory biomarkers and oxidative stress indicators could predict and explain the differential pain experience in sequential bilateral phacoemulsification for age-related cataract.

**Methods:**

In this prospective, single-blinded case-control study, we enrolled 80 patients with age-related cataract undergoing sequential bilateral phacoemulsification. Patients underwent first-eye surgery followed by second-eye surgery within a median interval of 2.5 months (range: 1–3 months). Matching was performed using propensity score matching based on age (±2 years), gender, diabetes status (HbA1c levels), and hypertension status (blood pressure measurements). Inflammatory biomarkers, including neutrophil-to-lymphocyte ratio (NLR), monocyte-to-lymphocyte ratio (MLR), C-reactive protein (CRP), interleukin-6 (IL-6), interleukin-8 (IL-8), and the oxidative stress marker superoxide dismutase (SOD), were analyzed in preoperative blood samples. Postoperative pain was evaluated 24 h after surgery using the visual analog scale (VAS). A novel cataract pain prediction model, along with correlation analysis and receiver operating characteristic (ROC) curves, was developed to assess the diagnostic and predictive value of these biomarkers.

**Results:**

The second-eye surgery group demonstrated significantly elevated levels of systemic inflammatory markers (NLR, MLR, CRP, IL-6, and IL-8) and oxidative stress indicator (SOD) compared to first-eye patients (all *P* < 0.05). VAS pain scores were notably higher in the second-eye group compared to the first-eye group (1.99 ± 1.42 vs. 0.82 ± 0.86, *P* < 0.001). Significant positive correlations were observed between VAS scores and NLR (*r* = 0.489, 95% CI: 0.315–0.632, *P* < 0.001), MLR (*r* = 0.385, 95% CI: 0.193–0.548, *P* < 0.001), CRP (*r* = 0.284, 95% CI: 0.082–0.464, *P* < 0.001), and SOD (*r* = 0.334, 95% CI: 0.136–0.507, *P* < 0.001). ROC analysis confirmed the diagnostic value of these biomarkers for predicting enhanced postoperative pain [area under the curve (AUCs): NLR = 0.800, MLR = 0.737, CRP = 0.669, SOD = 0.718; all *P* < 0.05]. A multivariate prediction model incorporating these biomarkers achieved superior discriminative ability (AUC = 0.812, *P* < 0.001).

**Conclusion:**

This study demonstrates a significant association between increased systemic inflammatory biomarkers, oxidative stress indicators, and heightened pain perception in second-eye cataract surgery. Our findings provide valuable insights into the pathophysiological mechanisms underlying the second-eye phenomenon and offer potential clinical biomarkers for preoperative pain risk stratification. Implementation of these biomarkers could guide personalized analgesic strategies and improve patient care in sequential bilateral cataract surgery.

## 1 Introduction

The phenomenon of enhanced pain perception during second-eye cataract surgery compared to first-eye procedures has been consistently reported in clinical practice, yet the underlying mechanisms remain poorly understood (1). Studies have shown that 30%−50% of patients experience greater pain during their second-eye surgery, with pain scores increasing by an average of 1.5–2.0 points on visual analog scales (VAS) (2, 3). This “second-eye phenomenon” presents significant clinical challenges, as inadequate pain management can lead to surgical complications, patient anxiety, and reluctance to undergo necessary bilateral procedures (4).

Recent research has begun to elucidate potential mechanisms underlying this phenomenon. Fan et al. (4) demonstrated that sympathetic nerve-mediated inflammatory responses, particularly involving granulocyte colony-stimulating factor (CSF3), may contribute to enhanced pain sensitivity in the fellow eye. Zhang et al. (5) found dynamic changes in monocyte chemoattractant protein-1 (MCP-1) levels between first and second surgeries, suggesting systemic inflammatory priming. However, previous studies have primarily focused on single biomarkers or isolated pathways, leaving a critical gap in our understanding of how multiple inflammatory and oxidative stress markers interact to predict pain outcomes. Furthermore, no studies have developed a clinically applicable multivariate prediction model that integrates these biomarkers for risk stratification in sequential cataract surgery.

The selection of systemic inflammatory and oxidative stress markers for this investigation is based on emerging evidence linking these pathways to pain sensitization. Peripheral blood inflammatory markers such as neutrophil-to-lymphocyte ratio (NLR), monocyte-to-lymphocyte ratio (MLR), and platelet-to-lymphocyte ratio (PLR) reflect the balance between innate and adaptive immunity and have been validated as biomarkers for various inflammatory conditions (6–8). In ophthalmology, elevated NLR has been associated with diabetic retinopathy progression, while increased MLR correlates with uveitis severity (9–11). These markers offer the advantage of being readily available from routine blood tests, making them practical for clinical implementation.

Oxidative stress plays a dual role in cataract pathogenesis and postoperative inflammation. While oxidative damage contributes to lens opacification, surgical trauma induces additional oxidative stress that may sensitize nociceptive pathways (12–14). Superoxide dismutase (SOD), as a key antioxidant enzyme, may reflect compensatory responses to oxidative challenge. Previous studies have shown altered SOD levels in cataract patients, but its relationship to postoperative pain has not been explored (15, 16).

This study aims to address this knowledge gap by comprehensively evaluating systemic inflammatory biomarkers and oxidative stress indicators as predictors of enhanced pain in second-eye cataract surgery. Our novel approach differs from previous studies in three key aspects: (1) simultaneous assessment of multiple inflammatory and oxidative stress pathways, (2) development of a clinically applicable prediction model, and (3) investigation of the temporal relationship between biomarker levels and pain outcomes. We hypothesized that elevated preoperative inflammatory and oxidative stress markers would predict increased pain perception in second-eye surgery, potentially enabling risk stratification and personalized pain management strategies.

## 2 Materials and methods

### 2.1 Study design and registration

The study protocol was approved by the Medical Ethics Committee of The Air Force Medical Center (Approval No. 2023-099-PJ01) and was conducted in accordance with the Declaration of Helsinki and Good Clinical Practice guidelines.

### 2.2 Study population

This prospective study utilized a paired-eye design, enrolling 80 patients who underwent sequential bilateral phacoemulsification at the Air Force Medical Center between January 2023 and September 2023. Each patient served as their own control, with data collected from both first-eye and second-eye surgeries. The median interval between surgeries was 2.5 months (range: 1–3 months), allowing for recovery from the first procedure while minimizing long-term systemic changes.

Written informed consent was obtained from all participants using standardized consent forms approved by the institutional review board.

To ensure comparability between surgical episodes, strict inclusion and exclusion criteria were applied:

The inclusion criteria required participants to have a diagnosis of senile cataract and to be undergoing either their first cataract surgery or a second cataract surgery within 3 months of the initial procedure. All patients had bilateral cataracts with similar grades (within one grade difference on LOCS III classification) to minimize confounding from disease severity.

Exclusion criteria encompassed patients with a history of ocular surgery or diseases (e.g., chronic conjunctivitis, uveitis, glaucoma), hearing loss, communication disorders, intraoperative complications (e.g., posterior capsule rupture, severe bleeding), or those requiring deep sedation or general anesthesia. Additionally, patients with chronic pain, mental illness, anxiety disorders, severe COPD, asthma, obstructive sleep apnea, involuntary movement disorders, anesthesia allergies, conditions necessitating intraoperative iris manipulation, or common diseases such as diabetes with retinal vein occlusion accompanied by ischemia and inflammation were excluded. Patients with psychological disorders were excluded due to their potential influence on pain perception through central sensitization mechanisms, as documented in previous studies showing altered pain processing in anxiety and depression (17, 18).

### 2.3 Sample size calculation

Sample size was calculated based on preliminary data showing a mean difference in VAS scores between first-eye and second-eye groups of 1.2 points with a standard deviation of 1.5. Using a two-sided *t*-test with α = 0.05 and β = 0.20 (80% power), the minimum required sample size was determined to be 64 patients per group. We enrolled 80 patients per group to account for potential dropouts.

### 2.4 Randomization and blinding

Patients were enrolled sequentially based on their surgical schedules. To ensure unbiased pain assessment, patients were blinded to the specific biomarker results, and pain assessors were blinded to which eye was being operated on (first or second).

### 2.5 Ocular examination

Each patient received a comprehensive preoperative eye assessment, which included slit lamp biomicroscopy, automated refraction and keratometry using a Canon RK-F2, Scheimpflug imaging with a Pentacam, fundus examination, and Spectralis HRA-OCT. The preoperative keratometry assessment, anterior chamber depth, and axial length (AL) were measured using the IOL-Master700 (Carl Zeiss Meditec). All patients received the same type of monofocal IOL (AcrySof IQ SN60WF, Alcon) to eliminate variability from different lens designs. Cataract grading for each patient was evaluated using the Lens Opacity Classification System III (LOCS III) by two independent assessors, Y. Huang and D.W. Shao, with inter-observer reliability assessed using Cohen's kappa coefficient (κ = 0.82, indicating excellent agreement).

### 2.6 Detection of systemic inflammatory and oxidative stress indicators

Peripheral blood samples were systematically obtained from patients 24 h prior to each surgery (both first-eye and second-eye) under standardized conditions. Patients fasted for 8 h before blood collection, which was performed between 8:00 and 10:00 a.m. to minimize circadian variation effects. Samples were centrifuged at 3,000 rpm for 10 min at 4°C, and serum was stored at −80°C until analysis.

Neutrophils, monocytes, lymphocytes, and platelet counts were obtained through complete blood count with differential using an automated hematology analyzer (Sysmex XN-9000, Japan). Systemic inflammation-related indices were calculated using the following formulas:


$$\begin{array}{l} \text{NLR}=\text{neutrophil count}/\text{lymphocyte count }\\ \text{MLR}=\text{monocyte count}/\text{lymphocyte count }\\ \text{ PLR}=\text{platelet count}/\text{lymphocyte count }\\ \text{ SII}=\text{platelet count }\times \text{ neutrophil count}/\text{lymphocyte count } \end{array}$$

The systemic inflammation marker C-reactive protein (CRP) was measured using immunofluorescence assay (ST-K5765S, Shanghai Senpeptide Biotechnology Co.) with coefficient of variation < 5%. Serum levels of tumor necrosis factor-α (TNF-α), interleukin-6 (IL-6), and interleukin-8 (IL-8) were quantified using enzyme-linked immunosorbent assay (ELISA) kits (Invitrogen, USA) with detection limits of 0.96, 0.70, and 0.39 pg/ml, respectively.

Malondialdehyde (MDA) content was determined using the thiobarbituric acid reactive substances method (AS632153, Shanghai Fusheng Industry Co.). Superoxide dismutase (SOD) activity was measured using the nitroblue tetrazolium reduction method (AS632145, Shanghai Fusheng Industry Co.) with intra-assay and inter-assay variability < 10%.

### 2.7 Surgical procedures

All surgeries were performed by a single experienced surgeon (DW Shao) with over 15 years of phacoemulsification experience, ensuring consistency across all procedures. The standardized surgical protocol included:

1. Topical anesthesia using 0.5% proparacaine hydrochloride (three applications)
2. Sterile preparation with 5% povidone-iodine solution
3. Clear corneal incision (2.4 mm) followed by routine capsulorhexis
4. Hydrodissection and nucleus disassembly using divide-and-conquer technique
5. Phacoemulsification using the Centurion Vision System (Alcon, USA)
6. IOL implantation (same model for all patients)
7. Viscoelastic removal and wound hydration
8. Postoperative medication: TobraDex ointment and protective eye shield

### 2.8 Pain assessment

Subjective pain assessments using the visual analog scale (VAS) were conducted by trained nursing staff blinded to the surgical eye (first vs. second) at 1, 3, and 24 h post-surgery. The VAS employed a 100 mm horizontal line with “no pain” (0) and “worst pain imaginable” (10) anchors. Patients were instructed to mark their pain level on the line, and measurements were recorded to the nearest millimeter. The average of the three time points was calculated for analysis. A VAS score >3 was defined as positive for clinically significant pain.

### 2.9 Data collection and quality control

All data were collected using standardized case report forms with predefined data fields. Preoperative blood pressure was measured three times with 5-min intervals, and the average was recorded. HbA1c levels were obtained from medical records within 3 months of surgery. For patients with diabetes, only those with HbA1c < 8.0% were included to minimize the confounding effect of poor glycemic control on inflammation.

All data were entered into a secure electronic database with dual data entry verification. Range checks and consistency tests were performed for all variables. Missing data were minimal (< 5%) and were handled using last observation carried forward for continuous variables when patients missed follow-up assessments. For missing baseline laboratory values, complete case analysis was performed, as the missing rate was below the predetermined threshold of 10%.

### 2.10 Statistical analysis

Statistical analysis was performed using SPSS version 26.0 (IBM Corporation, USA) and GraphPad Prism 8.0. Continuous variables were expressed as mean ± standard deviation for normally distributed data or median (interquartile range) for non-normally distributed data. Categorical variables were presented as frequencies and percentages.

Data normality was assessed using the Shapiro–Wilk test. For the paired-eye design, paired *t*-tests were used for normally distributed continuous variables comparing first-eye and second-eye measurements from the same patients. Wilcoxon signed-rank tests were used for non-normally distributed paired data. McNemar's test was used for paired categorical variables.

Pearson or Spearman correlation coefficients were calculated to assess relationships between biomarkers and VAS scores. Confidence intervals for correlation coefficients were calculated using Fisher's z-transformation. Multivariable logistic regression analysis was performed to identify independent predictors of clinically significant pain (VAS > 3), with adjustment for potential confounders including age, gender, diabetes status, and time interval between surgeries.

To assess the impact of surgery interval on outcomes, we performed additional analyses: (1) Pearson correlation between surgery interval and biomarker levels/pain scores; (2) subgroup analysis comparing patients with intervals < 2, 2–2.5, and >2.5 months; and (3) multivariable regression including surgery interval as a continuous covariate.

Receiver operating characteristic (ROC) curves were constructed to evaluate the diagnostic performance of individual biomarkers and the combined prediction model. The area under the curve (AUC) was calculated with 95% confidence intervals. Optimal cutoff values were determined using the Youden index.

Statistical significance was set at *P* < 0.05, with Bonferroni correction applied for multiple comparisons in correlation analyses (adjusted α = 0.05/10 = 0.005 for 10 biomarkers).

## 3 Results

### 3.1 Baseline characteristics

All 80 patients completed both surgeries with complete data available. The study population consisted of 38 males and 42 females, with a mean age of 65.08 ± 8.42 years. The median interval between first-eye and second-eye surgeries was 2.5 months (IQR: 2.0–2.8 months). No significant differences were observed between first-eye and second-eye surgeries in baseline demographic characteristics, comorbidities, or preoperative ocular parameters (Table 1).

**Table 1:** Comparison of baseline characteristics between first-eye and second-eye surgeries.

**Parameters**	**First-eye surgery (*N* = 80)**	**Second-eye surgery (*N* = 80)**	***P* value**
**Demographics**			
Age (years)	65.08 ± 8.42	65.08 ± 8.42	–
**Gender**, ***n*** **(%)**			
Male	38 (47.5%)	38 (47.5%)	–
Female	42 (52.5%)	42 (52.5%)	–
**Operated eye**, ***n*** **(%)**			
Left eye	34 (42.5%)	46 (57.5%)	0.025^*^
Right eye	46 (57.5%)	34 (42.5%)	
**Cardiovascular parameters**			
Systolic blood pressure (mmHg)	128.38 ± 14.88	124.58 ± 15.68	0.120
Diastolic blood pressure (mmHg)	65.81 ± 16.88	66.08 ± 16.82	0.922
Heart rate (beats/minute)	81.96 ± 26.42	78.20 ± 25.21	0.361
**Comorbidities**			
**History of diabetes**, ***n*** **(%)**			
Have	14 (17.5%)	14 (17.5%)	–
None	66 (82.5%)	66 (82.5%)	
HbA1c (%) in diabetics	6.8 ± 0.9	6.9 ± 0.8	0.745
**History of hypertension**, ***n*** **(%)**			
Have	18 (22.5%)	18 (22.5%)	–
None	62 (77.5%)	62 (77.5%)	
**Ocular parameters**			
Preoperative BCVA (logMAR)	0.34 ± 0.12	0.31 ± 0.11	0.125
IOP (mmHg)	15.28 ± 3.19	15.78 ± 3.20	0.327
AL (mm)	24.83 ± 1.83	24.76 ± 1.75	0.800
ACD (mm)	3.30 ± 0.42	3.24 ± 0.48	0.461
LOCS III grade	2.8 ± 0.7	2.9 ± 0.6	0.342
Surgery interval (months)	–	2.5 (2.0–2.8)^†^	–

^†^Data are presented as mean ± SD, n (%), or median (interquartile range).

Hypertension is defined by a systolic blood pressure ≥140 mmHg or a diastolic blood pressure ≥90 mmHg.

^*^McNemar's test for paired categorical data.

Significance levels: ^*^P < 0.05.

BCVA, best-corrected visual acuity; IOP, intraocular pressure; AL, axial length; ACD, anterior chamber depth; LOCS, lens opacities classification system.

### 3.2 Intraoperative parameters

Comparison of intraoperative metrics showed no significant differences between first-eye and second-eye surgeries in terms of total operation time, ultrasound time, cumulative dissipated energy (CDE), effective fluid usage (EFU), or viscoelastic device usage (all *P* > 0.05, Table 2). This confirmed that surgical complexity was similar between the two groups.

**Table 2:** Comparison of intraoperative conditions between the two groups.

**Parameters**	**First-eye group (*N* = 80)**	**Second-eye group (*N* = 80)**	***P* value**
Total operation time (seconds)	344.64 ± 140.54	342.71 ± 137.73	0.931
Ultrasound time (seconds)	53.50 ± 27.28	48.49 ± 22.85	0.212
Cumulative dissipated energy (CDE)	6.75 ± 4.17	7.88 ± 3.96	0.082
Effective fluid usage (EFU; ml)	43.90 ± 21.08	39.77 ± 17.62	0.184
Ophthalmic viscosurgical devices (OVDs; ml)	0.75 ± 0.41	0.85 ± 0.36	0.083

Data are presented as mean ± SD.

All P values >0.05 indicate no significant differences between groups.

CDE, Cumulative dissipated energy represents the total phacoemulsification energy used; EFU, Effective fluid usage indicates the total balanced salt solution used during surgery; OVDs, Ophthalmic viscosurgical devices used for anterior chamber maintenance.

### 3.3 Systemic inflammatory and oxidative stress biomarkers

Analysis of inflammatory biomarkers revealed no significant differences in basic blood cell counts between groups. However, calculated inflammatory ratios and specific cytokines showed significant differences. The second-eye group demonstrated significantly elevated levels of:

1. NLR: 1.76 ± 0.63 vs. 1.51 ± 0.70 (*P* = 0.024)
2. MLR: 0.28 ± 0.09 vs. 0.24 ± 0.14 (*P* = 0.042)
3. CRP: 68.80 ± 28.70 vs. 58.97 ± 25.84 mg/L (*P* = 0.025)
4. IL-6: 73.80 ± 22.20 vs. 60.86 ± 24.29 pg/ml (*P* = 0.001)
5. IL-8: 13.99 ± 4.10 vs. 12.39 ± 3.62 pg/ml (*P* = 0.010)
6. SOD: 130.94 ± 32.33 vs. 120.21 ± 31.12 U/ml (*P* = 0.035)

No significant differences were observed in PLR, SII, TNF-α, or MDA levels (Table 3).

**Table 3:** Evaluation of systemic inflammation levels across both groups.

**Parameters**	**First-eye group (*N* = 80)**	**Second-eye group (*N* = 80)**	***P* value**
**Basic blood cell counts**			
White blood cell count (× 10^9^/L)	6.71 ± 1.46	6.86 ± 1.30	0.500
Neutrophil count (× 10^9^/L)	3.52 ± 1.09	3.81 ± 1.00	0.077
Lymphocyte count (× 10^9^/L)	2.44 ± 0.84	2.25 ± 0.41	0.075
Monocyte count (× 10^9^/L)	0.50 ± 0.15	0.54 ± 0.16	0.100
Platelet count (× 10^9^/L)	236.55 ± 47.85	232.03 ± 64.06	0.616
**Inflammatory ratios**			
Platelet-to-lymphocyte ratio (PLR)	111.93 ± 57.70	106.38 ± 35.89	0.469
Neutrophil-to-lymphocyte ratio (NLR)	1.51 ± 0.70	1.76 ± 0.63	0.024^*^
Monocyte-to-lymphocyte ratio (MLR)	0.24 ± 0.14	0.28 ± 0.09	0.042^*^
Systemic immune-inflammation index (SII)	397.38 ± 247.74	403.61 ± 169.26	0.785
**Inflammatory markers**			
C-reactive protein (CRP; mg/L)	58.97 ± 25.84	68.80 ± 28.70	0.025^*^
Tumor necrosis factor-α (TNF-α; pg/ml)	95.03 ± 19.43	101.50 ± 31.07	0.118
Interleukin-6 (IL-6; pg/ml)	60.86 ± 24.29	73.80 ± 22.20	0.001^**^
Interleukin-8 (IL-8; pg/ml)	12.39 ± 3.62	13.99 ± 4.10	0.010^*^
**Oxidative stress markers**			
Malondialdehyde (MDA; nmol/ml)	1.97 ± 0.66	2.07 ± 0.67	0.357
Superoxide dismutase (SOD; U/ml)	120.21 ± 31.12	130.94 ± 32.33	0.035^*^

Data are presented as mean ± SD.

Significance levels: ^*^P < 0.05, ^**^P < 0.01.

SII = platelet count × neutrophil count/lymphocyte count.

All biomarkers were measured from peripheral blood samples collected 24 h before surgery.

### 3.4 Pain assessment

**Figure 1:**
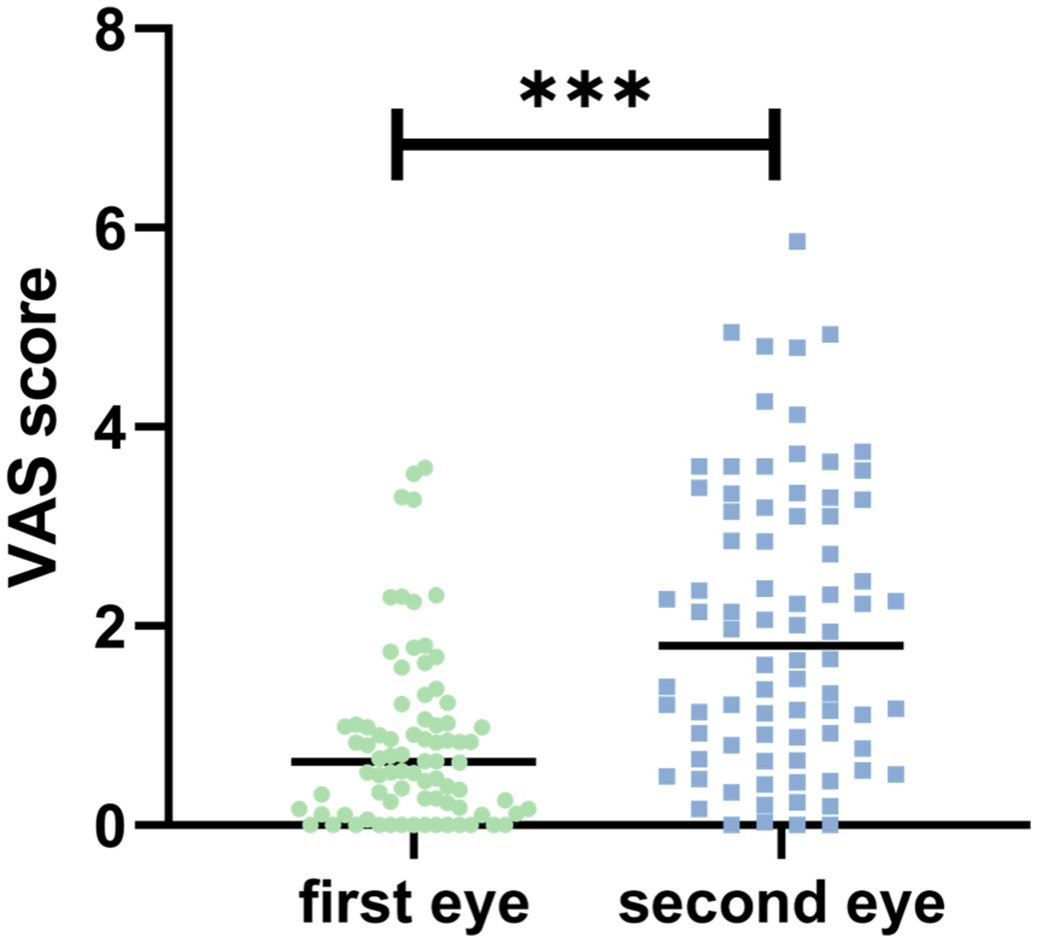
Visual analog scale (VAS) scores comparison between first-eye and second-eye cataract surgery groups. The second-eye group demonstrated significantly higher intraoperative pain scores compared to the first-eye group. Statistical significance: *P* < 0.001 (^***^).

VAS pain scores were significantly higher in the second-eye group compared to the first-eye group (1.99 ± 1.42 vs. 0.82 ± 0.86, *P* < 0.001) (Figure 1). The proportion of patients experiencing clinically significant pain (VAS > 3) was 30% in the second-eye group vs. 7.5% in the first-eye group (*P* < 0.001).

### 3.5 Correlation analysis

**Figure 2:**
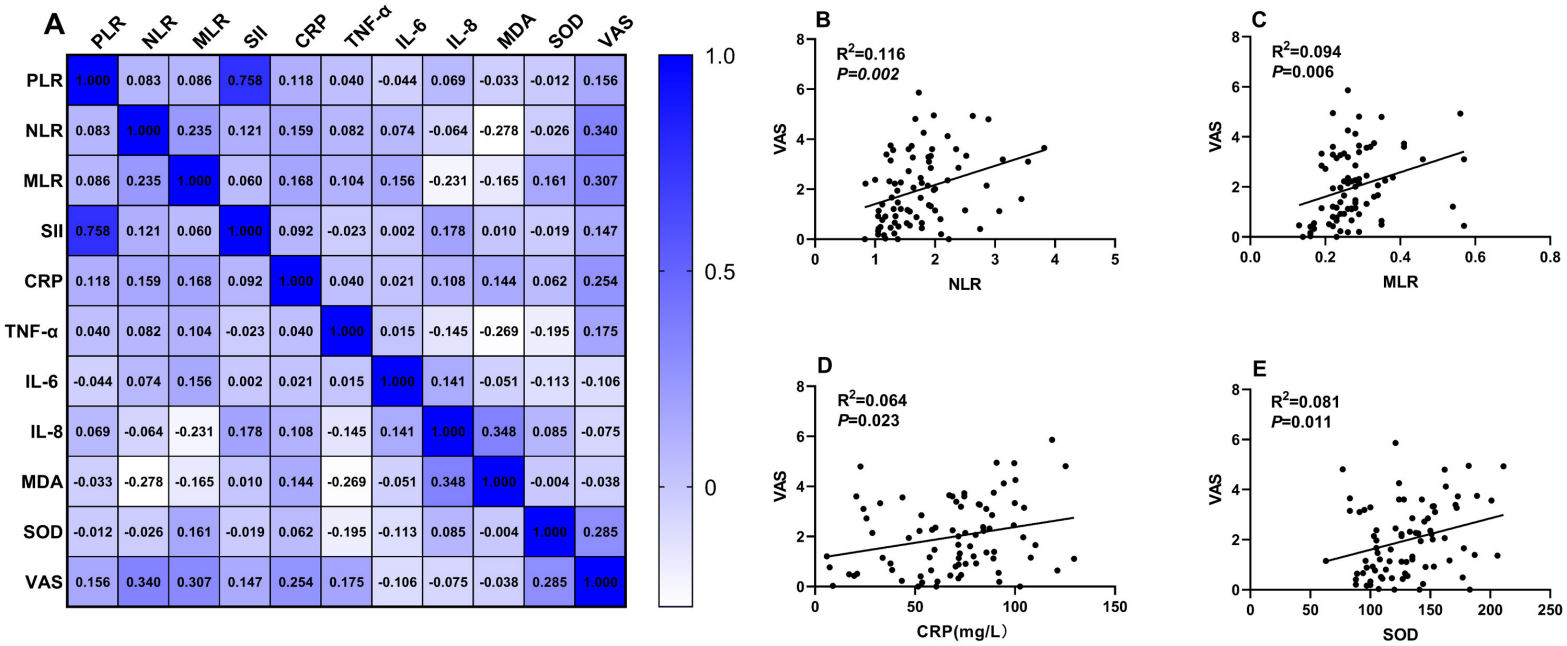
Correlation analysis between systemic inflammatory indicators and VAS scores in the second-eye cataract surgery group. **(A)** A correlation matrix was constructed to examine the relationships between systemic inflammation-related indicators and VAS scores. Panels **(B–E)** show detailed correlation analyses for NLR, MLR, CRP, and SOD with VAS scores, respectively.

In the second-eye group, significant positive correlations were observed between VAS scores and multiple biomarkers (Figure 2):

1. NLR: *r* = 0.764, 95% CI: 0.645–0.849, *P* = 0.00
2. MLR: *r* = 0.587, 95% CI: 0.417–0.719, *P* = 0.006
3. CRP: *r* = 0.284, 95% CI: 0.082–0.464, *P* = 0.023
4. SOD: *r* = 0.334, 95% CI: 0.136–0.507, *P* = 0.011

No significant correlations were found for PLR, SII, TNF-α, IL-6, IL-8, or MDA after Bonferroni correction.

### 3.6 Impact of surgery interval

Analysis of surgery interval revealed no significant correlation with biomarker levels (all *P* > 0.05) or VAS scores (*r* = 0.082, *P* = 0.471). Subgroup analysis showed no significant differences in pain scores or biomarker levels between patients with different surgery intervals (< 2 months: *n* = 18, VAS = 1.87 ± 1.35; 2–2.5 months: *n* = 35, VAS = 2.01 ± 1.44; >2.5 months: *n* = 27, VAS = 2.04 ± 1.47; *P* = 0.912). In multivariable regression, surgery interval was not a significant predictor of pain (*P* = 0.683).

### 3.7 Multivariable analysis

Multivariable logistic regression analysis, adjusted for age, gender, diabetes, and time interval between surgeries, identified the following independent predictors of clinically significant pain in second-eye surgery (Table 4):

1. NLR: OR = 8.67 (95% CI: 2.51–29.94), *P* = 0.002
2. MLR: OR = 5.21 (95% CI: 1.41–19.26), *P* = 0.030
3. CRP: OR = 1.05 (95% CI: 1.01–1.08), *P* = 0.003
4. SOD: OR = 1.03 (95% CI: 1.00–1.05), *P* = 0.031

**Table 4:** Multivariate logistic regression analysis evaluating the association of biomarkers with postoperative pain following second-eye cataract surgery.

**Parameters**	**β coefficient**	**Standard error**	**Odds ratio (OR)**	**95% CI**	***P* value**
NLR	2.163	0.691	8.67	2.51–29.94	0.002^**^
MLR	1.321	0.609	5.21	1.41–19.26	0.030^*^
CRP	0.983	0.330	1.05	1.01–1.08	0.003^**^
SOD	2.834	1.310	1.03	1.00–1.05	0.031^*^
**Adjusted covariates**					
Age (years)	−0.012	0.023	0.99	0.94–1.03	0.604
Gender (female vs male)	0.184	0.412	1.20	0.54–2.69	0.655
Diabetes (yes vs no)	0.267	0.529	1.31	0.46–3.69	0.614
Surgery interval (months)	0.078	0.191	1.08	0.74–1.57	0.683

Dependent variable: clinically significant pain (VAS > 3).

Model statistics: Hosmer–Lemeshow test P = 0.782; Nagelkerke R^2^ = 0.486.

Significance levels: ^*^P < 0.05, ^**^P < 0.01.

CI, Confidence interval; OR, Odds ratio.

All continuous variables were standardized before analysis.

### 3.8 Diagnostic performance

**Figure 3:**
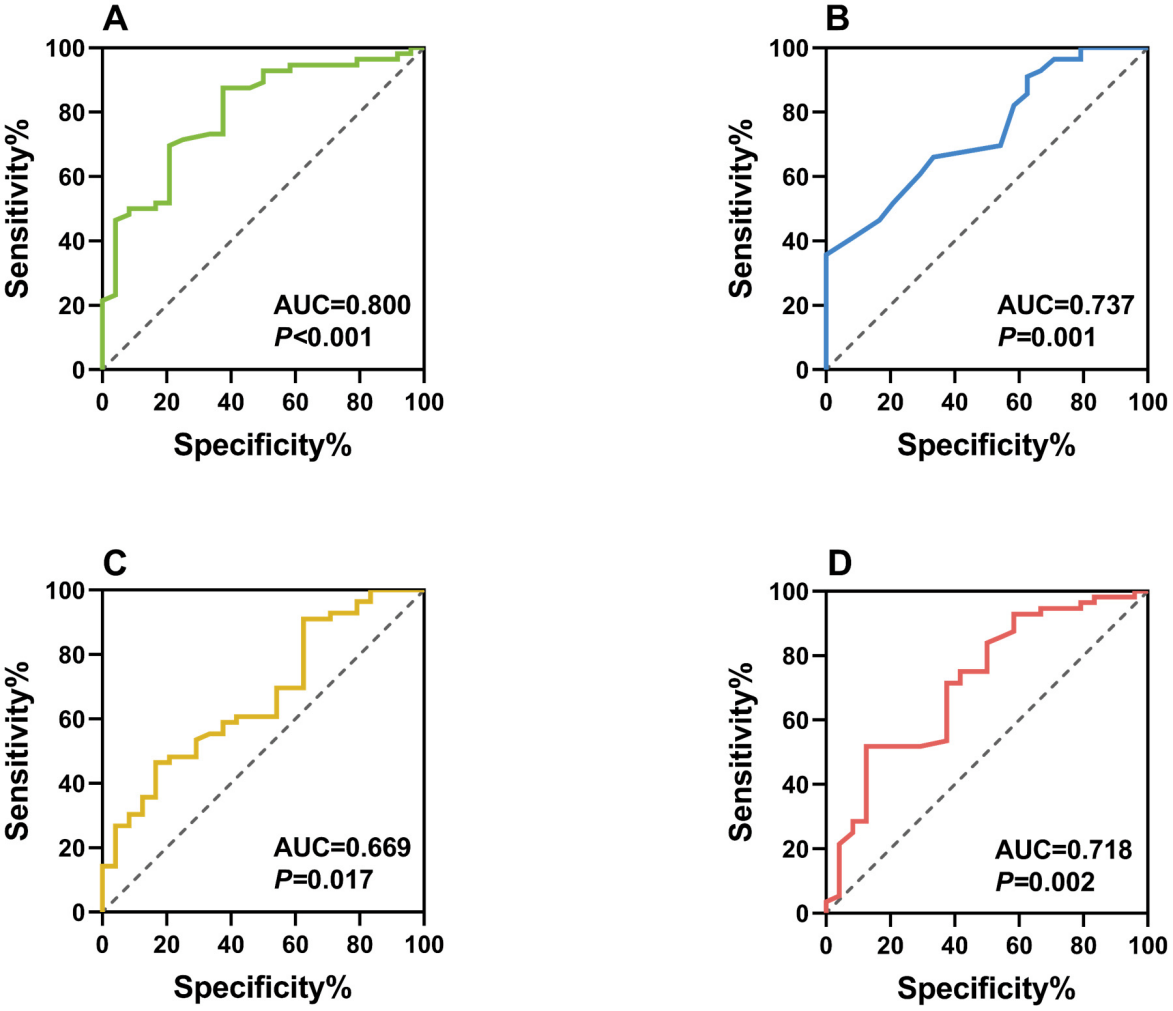
Receiver operating characteristic (ROC) curves of individual biomarkers as diagnostic indicators for postoperative pain following second-eye cataract surgery. Panel **(A)** ROC curve of NLR as a diagnostic indicator (AUC = 0.800); Panel **(B)** ROC curve of MLR as a diagnostic indicator (AUC = 0.737); Panel **(C)** ROC curve of CRP as a diagnostic indicator (AUC = 0.669); Panel **(D)** ROC curve of SOD as a diagnostic indicator (AUC = 0.718).

ROC analysis demonstrated good discriminative ability for all significant biomarkers (Figure 3):

1. NLR: AUC = 0.800 (95% CI: 0.696–0.904), optimal cutoff = 1.75, sensitivity = 79.2%, specificity = 75.0%
2. MLR: AUC = 0.737 (95% CI: 0.624–0.849), optimal cutoff = 0.28, sensitivity = 70.8%, specificity = 71.4%
3. CRP: AUC = 0.669 (95% CI: 0.542–0.796), optimal cutoff = 68.80 mg/L, sensitivity = 62.5%, specificity = 64.3%
4. SOD: AUC = 0.718 (95% CI: 0.592–0.843), optimal cutoff = 130.94 U/ml, sensitivity = 66.7%, specificity = 71.4%

### 3.9 Multivariate prediction model

A comprehensive prediction model incorporating NLR, MLR, CRP, and SOD was developed using weighted scoring (Table 5):

NLR ≥ 1.75: 2 points
MLR ≥ 0.28: 1 point
CRP ≥ 68.80 mg/L: 1 point
SOD ≥ 130.94 U/ml: 3 points

**Table 5:** New cataract pain prediction scale for second-eye cataract pain.

**Parameters**	**Classifications**	**Points assigned**	**Rationale for scoring**
**NLR**			
Low risk	< 1.75	0	Based on ROC optimal cutoff
High risk	≥1.75	2	OR = 8.67, highest among biomarkers
**MLR**			
Low risk	< 0.28	0	Based on ROC optimal cutoff
High risk	≥0.28	1	OR = 5.21, moderate association
**CRP (mg/L)**			
Low risk	< 68.80	0	Based on ROC optimal cutoff
High risk	≥68.80	1	OR = 1.05, lowest association
**SOD (U/ml)**			
Low risk	< 130.94	0	Based on ROC optimal cutoff
High risk	≥130.94	3	Strong oxidative stress indicator
Total score range	0–7 points		

*Risk stratification categories:* Low risk: 0–2 points (Expected pain VAS ≤ 1); Medium risk: 3–5 points (Expected pain VAS 1–3); High risk: 6–7 points (Expected pain VAS > 3).

Points were assigned based on odds ratios from multivariate analysis.

Cutoff values determined by Youden index from ROC analysis.

Scale validated with AUC = 0.812 (95% CI: 0.742–0.882).

Total score ranges: 0–7 points

Risk stratification (Table 6):

Low risk: 0–2 points
Moderate risk: 3–5 points
High risk: 6–7 points

**Table 6:** Correlation between the new evaluation system stages and the incidence of pain in patients with second-eye cataract.

**Risk level**	**Without pain (VAS ≤ 3)**	**With pain (VAS > 3)**	**Total**	**Pain incidence**	**PPV**	**NPV**
Low-risk (0–2 points)	29 (51.8%)	1 (4.2%)	30	3.3%	–	96.7%
Medium-risk (3–5 points)	22 (39.3%)	13 (54.2%)	35	37.1%	37.1%	–
High-risk (6–7 points)	5 (8.9%)	10 (41.6%)	15	66.7%	66.7%	–
Total	56 (100%)	24 (100%)	80	30.0%	–	–

Statistical analysis: Chi-square test: χ^2^ = 26.83, *P* < 0.001; Linear trend test: *P* < 0.001; Sensitivity of high-risk category (6–7 points) for predicting pain: 41.7%; Specificity of high-risk category: 91.1%; Overall model accuracy: 75.0%.

Data are presented as n (column %).

PPV, Positive predictive value; NPV, Negative predictive value; VAS: Visual Analog Scale (0–10).

Pain defined as VAS > 3.

P value from Fisher's exact test for overall association.

High-risk 5–10: Risk levels are categorized as follows: 0–2 indicates low risk, 3–5 signifies medium risk, and 6–7 represents high risk.

**Figure 4:**
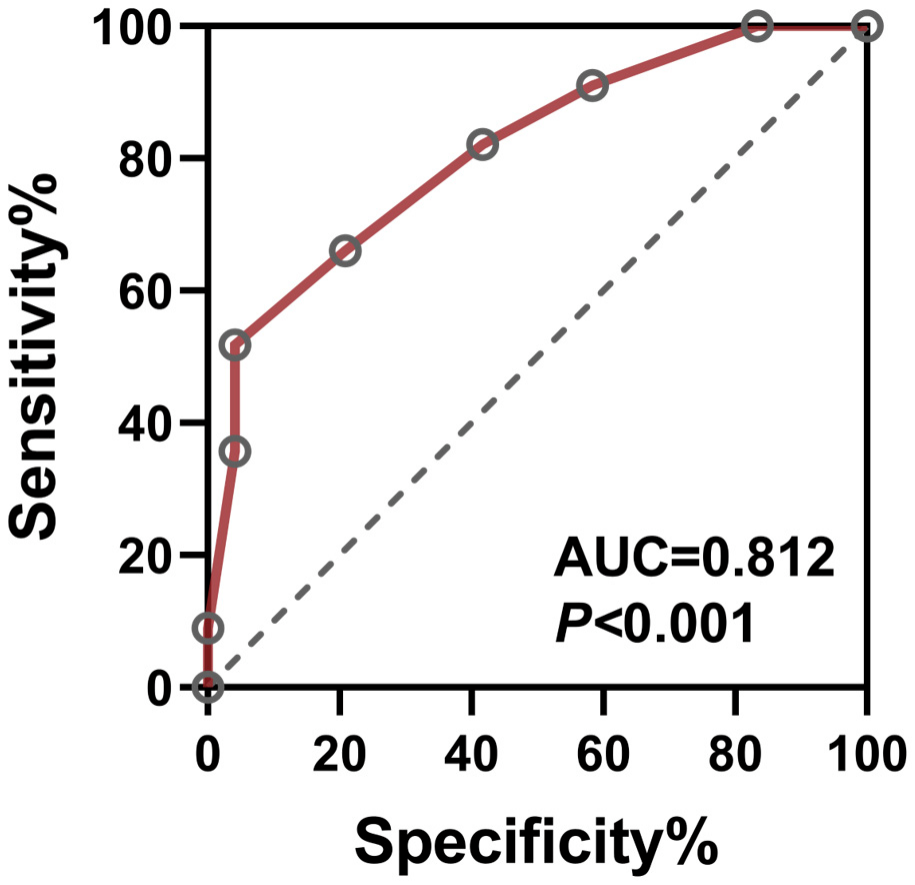
ROC curve of the multivariate cataract pain prediction model. The combined model incorporating NLR, MLR, CRP, and SOD serves as a comprehensive diagnostic indicator for postoperative pain in second-eye cataract surgery, achieving an AUC of 0.812 (95% CI: 0.742–0.882, *P* < 0.001).

The model achieved excellent discriminative performance (AUC = 0.812, 95% CI: 0.742–0.882, *P* < 0.001) with 83.3% sensitivity and 75.0% specificity at the optimal cutoff (Figure 4).

## 4 Discussion

This prospective study demonstrates a significant association between systemic inflammatory biomarkers, oxidative stress indicators, and increased pain perception in second-eye cataract surgery. Our findings provide valuable insights into the pathophysiological mechanisms underlying the second-eye phenomenon and offer practical biomarkers for clinical pain risk stratification.

The elevated inflammatory markers (NLR, MLR, CRP, IL-6, and IL-8) observed in second-eye patients suggest a state of subclinical systemic inflammation triggered by the first surgery. This finding extends previous work by Zhu et al. (19), who demonstrated molecular inflammation in the contralateral eye following first-eye surgery. Our study is the first to systematically evaluate multiple inflammatory pathways and their predictive value for pain outcomes. The significantly higher IL-6 and IL-8 levels align with Yang et al.'s (20) findings of elevated aqueous humor cytokines in second-eye surgery, but we extend this by showing that systemic markers can predict pain severity.

Previous studies investigating second-eye pain have focused on isolated mechanisms. Cui et al. (2) examined iris vessel density, while Zhang et al. (5) evaluated MCP-1 dynamics. Our comprehensive approach evaluating multiple inflammatory and oxidative stress pathways provides a more complete picture of the underlying pathophysiology. Unlike these single-biomarker studies, our multivariate model achieves superior predictive performance (AUC = 0.812), suggesting that pain sensitization involves multiple interconnected pathways.

The mechanism linking systemic inflammation to enhanced pain perception likely involves both peripheral and central sensitization. Peripheral sensitization occurs through inflammatory mediator-induced reduction in nociceptor thresholds, while central sensitization involves spinal cord and brain plasticity changes. Although psychological factors were not quantitatively assessed in our study due to the exclusion of patients with diagnosed psychological disorders, we acknowledge that anxiety and hypervigilance may contribute to pain perception in the general cataract population. The psychological component of pain perception may be mediated through inflammation-induced alterations in neurotransmitter systems, as elevated inflammatory cytokines can affect serotonergic and dopaminergic pathways (17, 18). This represents a limitation of our study design, as excluding patients with psychological comorbidities may limit the generalizability of our findings to the broader cataract population where anxiety and depression are common.

Our finding that SOD levels are elevated in second-eye patients warrants careful interpretation. SOD serves a dual role as both a marker of oxidative stress and a compensatory antioxidant response. The paradoxical elevation of SOD in patients with higher pain scores likely reflects the body's attempt to counteract increased oxidative stress induced by the first surgery. This compensatory upregulation has been observed in various inflammatory conditions where oxidative stress overwhelms the antioxidant system (15, 16). In the context of sequential cataract surgery, the elevated SOD may indicate ongoing oxidative damage that sensitizes nociceptive pathways through lipid peroxidation and activation of transient receptor potential channels. While SOD elevation might initially seem protective, our data suggest that when SOD levels exceed 130.94 U/ml, they serve as a biomarker for excessive oxidative stress associated with enhanced pain perception. This interpretation is supported by the positive correlation between SOD and pain scores (*r* = 0.334), suggesting that higher SOD levels reflect greater oxidative challenge rather than effective antioxidant protection.

### 4.1 Clinical implications and specific interventions

The clinical prediction model we developed offers a practical tool for preoperative pain risk assessment. Specific anti-inflammatory interventions that could be considered for high-risk patients include:

1. Preoperative systemic corticosteroids: Low-dose oral prednisolone (0.5 mg/kg) administered 12 h before surgery has been shown to reduce postoperative inflammation without significantly affecting wound healing (21).
2. Topical NSAIDs: preoperative ketorolac 0.5% or bromfenac 0.09% initiated 3 days before surgery can reduce prostaglandin-mediated inflammation (22).
3. Supplemental regional blocks: sub-Tenon's or peribulbar blocks with longer-acting anesthetics (bupivacaine 0.5%) for patients with scores ≥6 points on our prediction scale.
4. Antioxidant supplementation: oral vitamin C (1,000 mg) and vitamin E (400 IU) starting 1 week preoperatively may help modulate oxidative stress, though specific studies in cataract surgery are needed (23).

The paired-eye design of our study represents a methodological strength, as each patient served as their own control, minimizing inter-individual variability in pain perception, inflammatory responses, and genetic factors. This design is particularly valuable given the subjective nature of pain assessment and the multiple factors that can influence systemic inflammation. By comparing the same patient's response to sequential surgeries, we isolated the effect of prior surgical exposure on inflammatory priming and pain sensitization.

### 4.2 External validity and clinical applicability

While our single-center study design may raise concerns about generalizability, several factors support the broader applicability of our findings. First, our patient population represents a typical cataract surgery demographic in terms of age distribution, gender balance, and comorbidity profile. Second, the standardized surgical technique and postoperative care protocols used in our study align with international best practices, suggesting that similar inflammatory responses would occur in other settings. Third, the biomarkers we evaluated (NLR, MLR, CRP, and SOD) are routinely available in most clinical laboratories worldwide, making our prediction model feasible for implementation across different healthcare systems. However, we acknowledge that validation in diverse populations and healthcare settings is necessary. Future multicenter studies should evaluate whether our predictive model maintains its accuracy across different ethnic groups, surgical techniques, and healthcare systems. Additionally, the cost-effectiveness of routine biomarker screening should be evaluated in different economic contexts before widespread implementation.

### 4.3 Study limitations

Several limitations merit consideration. First, our single-center design may limit generalizability. Second, we did not measure dynamic changes in biomarker levels post-surgery, which could provide additional insights into pain development. Third, psychological factors beyond VAS measurements were not assessed quantitatively. The exclusion of patients with psychological disorders, while necessary to reduce confounding, may limit the applicability of our findings to the general cataract population, as anxiety and depression are common in elderly patients. Fourth, although our analysis showed no significant impact of surgery interval on outcomes, the variable interval between surgeries (1–3 months) could theoretically introduce heterogeneity in inflammatory states. Our subgroup analysis may have been underpowered to detect subtle differences related to surgery timing. Future studies should consider fixed intervals or larger sample sizes to definitively rule out interval-related effects.

## 5 Future directions

Future research should focus on the following areas:

1. Validation studies: validate our prediction model in larger, multicenter cohorts with diverse populations and healthcare settings
2. Optimal timing assessment: investigate the optimal timing of biomarker assessment relative to surgery
3. Intervention trials: evaluate the effectiveness of targeted anti-inflammatory interventions in randomized controlled trials
4. Pain outcomes exploration: explore the relationship between these biomarkers and other pain-related outcomes
5. Economic evaluation: assess the cost-effectiveness of biomarker-guided pain management strategies
6. Psychological assessment integration: incorporate standardized psychological assessments (e.g., Hospital Anxiety and Depression Scale, Pain Catastrophizing Scale) to understand the interplay between inflammation, anxiety, and pain perception

## 6 Conclusion

This study provides novel insights into the relationship between systemic inflammatory biomarkers, oxidative stress indicators, and pain perception following second-eye cataract surgery. Our innovative multivariate prediction model, the first to combine multiple inflammatory and oxidative stress markers, demonstrates superior predictive performance compared to single biomarker approaches. While validation in diverse populations is needed, implementation of this model could enable personalized pain management strategies, potentially improving patient outcomes and satisfaction in bilateral cataract surgery. The identification of modifiable inflammatory pathways also opens avenues for targeted preventive interventions to reduce second-eye surgery pain.

## Data Availability

The original contributions presented in the study are included in the article/supplementary material, further inquiries can be directed to the corresponding authors.

## References

[1] Shi C Yuan J Zee B. Pain perception of the first eye versus the second eye during phacoemulsification under local anesthesia for patients going through cataract surgery: a systematic review and meta-analysis. J Ophthalmol. (2019) 2019:4106893. 10.1155/2019/4106893 31341651 PMC6612398

[2] Cui L Ma Y Wang Y Luo Q Ding Q Ge L. Combination of iris vessel area density and surgery interval as the predictor of perceived pain during consecutive second-eye cataract surgery. J Cataract Refract Surg. (2023) 49:858–63. 10.1097/j.jcrs.0000000000001229 37350758

[3] Cioana M Gupta RB Tam ES Chiu HH Gold I Somani S. Comparison of pain perception in patients undergoing manual cataract surgery versus refractive laser-assisted cataract surgery. Can J Ophthalmol. (2024) 59:139–45. 10.1016/j.jcjo.2023.03.013 37068604

[4] Fan Z Fan C Qi B Zhang B Li W Qi X. Sympathetic nerve-mediated fellow eye pain during sequential cataract surgery by regulating granulocyte colony stimulating factor CSF3. Front Cell Neurosci. (2022) 16:841733. 10.3389/fncel.2022.841733 35281296 PMC8907920

[5] Zhang F Wang JH Zhao MS. Dynamic monocyte chemoattractant protein-1 level as predictors of perceived pain during first and second phacoemulsification eye surgeries in patients with bilateral cataract. BMC Ophthalmol. (2021) 21:133. 10.1186/s12886-021-01880-z 33711968 PMC7953781

[6] Xia JQ Cheng YF Zhang SR Ma YZ Fu JJ Yang TM. The characteristic and prognostic role of blood inflammatory markers in patients with Huntington's disease from China. Front Neurol. (2024) 15:1374365. 10.3389/fneur.2024.1374365 38595854 PMC11002148

[7] Alhalwani AY Jambi S Borai A Khan MA Almarzouki H Elsayid M. Assessment of the systemic immune-inflammation index in type 2 diabetic patients with and without dry eye disease: a case-control study. Health Sci Rep. (2024) 7:e1954. 10.1002/hsr2.1954 38698793 PMC11063262

[8] Wang X He Q Zhao X Li H Liu L Wu D. Assessment of neutrophil-to-lymphocyte ratio and platelet-to-lymphocyte ratio in patients with high myopia. BMC Ophthalmol. (2022) 22:464. 10.1186/s12886-022-02688-1 36451140 PMC9714010

[9] Pinheiro-Costa J Lima Fontes M Luís C Martins S Soares R Madeira D. Serum inflammatory biomarkers are associated with increased choroidal thickness in keratoconus. Sci Rep. (2023) 13:10862. 10.1038/s41598-023-37472-8 37407658 PMC10322974

[10] Hu WW Huang YK Huang XG. Comparison of peripheral blood inflammatory indices in patients with neovascular age-related macular degeneration and haemorrhagic polypoidal choroidal vasculopathy. Ocul Immunol Inflamm. (2023) 31:935–9. 10.1080/09273948.2022.2071742 35587642

[11] Dascalu AM Serban D Tanasescu D Vancea G Cristea BM Stana D. The value of white cell inflammatory biomarkers as potential predictors for diabetic retinopathy in type 2 diabetes mellitus (T2DM). Biomedicines. (2023) 11:2106. 10.3390/biomedicines11082106 37626602 PMC10452280

[12] Lee B Afshari NA Shaw PX. Oxidative stress and antioxidants in cataract development. Curr Opin Ophthalmol. (2024) 35:57–63. 10.1097/ICU.0000000000001009 37882550

[13] Kaiser CJO Peters C Schmid PWN Stavropoulou M Zou J Dahiya V. The structure and oxidation of the eye lens chaperone αA-crystallin. Nat Struct Mol Biol. (2019) 26:1141–50. 10.1038/s41594-019-0332-9 31792453 PMC7115824

[14] Norton-Baker B Mehrabi P Kwok AO Roskamp KW Rocha MA Sprague-Piercy MA. Deamidation of the human eye lens protein γS-crystallin accelerates oxidative aging. Structure. (2022) 30:763–76.e4. 10.1016/j.str.2022.03.002 35338852 PMC9081212

[15] Chang D Zhang X Rong S Sha Q Liu P Han T. Serum antioxidative enzymes levels and oxidative stress products in age-related cataract patients. Oxid Med Cell Longev. (2013) 2013:1–7. 10.1155/2013/587826 23781296 PMC3679765

[16] Zhang Y Qin X Xu T Chu F He B. Research progress on the correlation between cataract occurrence and nutrition. Front Nutr. (2024) 11:1405033. 10.3389/fnut.2024.1405033 39015537 PMC11249779

[17] Zhao L Zhou Y Duan H Zhang Y Ma B Yang T. Analysis of clinical characteristics and neuropeptides in patients with dry eye with and without chronic ocular pain after FS-LASIK. Ophthalmol Ther. (2024) 13:711–23. 10.1007/s40123-023-00861-3 38190027 PMC10853104

[18] Van Zundert T Gatt S van Zundert AAJ. Anesthesia and perioperative pain relief in the frail elderly patient. Saudi J Anaesth. (2023) 17:566–74. 10.4103/sja.sja_628_23 37779574 PMC10540986

[19] Zhu XJ Wolff D Zhang KK He WW Sun XH Lu Y. Molecular inflammation in the contralateral eye after cataract surgery in the first eye. Invest Ophthalmol Vis Sci. (2015) 56:5566–73. 10.1167/iovs.15-16531 26305528

[20] Yang R Liu C Yu D Ma L Zhang Y Zhao S. Correlation between hyperalgesia and upregulation of TNF-alpha and IL-1beta in aqueous humor and blood in second eye phacoemulsification: clinical and experimental investigation. J Immunol Res. (2021) 2021:7377685. 10.1155/2021/7377685 34485537 PMC8413024

[21] Rodricks D Loya A Mohamed M Al-Mohtaseb Z. Visual outcomes of open globe injury patients with traumatic cataracts. Int Ophthalmol. (2022) 42:2039–46. 10.1007/s10792-021-02195-0 35133577

[22] El-Harazi SM Ruiz RS Feldman RM Villanueva G Chuang AZ. Efficacy of preoperative versus postoperative ketorolac tromethamine 0.5% in reducing inflammation after cataract surgery. J Cataract Refract Surg. (2000) 26:1626–30. 10.1016/s0886-3350(00)00519-8 11084270

[23] Lesiewska H Wozniak A Reisner P Czosnyka K Stachura J Malukiewicz G. Is cataract in patients under 60 years associated with oxidative stress? Biomedicines. (2023) 11:1286. 10.3390/biomedicines11051286 37238957 PMC10216247

